# ProKnow: Process knowledge for safety constrained and explainable question generation for mental health diagnostic assistance

**DOI:** 10.3389/fdata.2022.1056728

**Published:** 2023-01-09

**Authors:** Kaushik Roy, Manas Gaur, Misagh Soltani, Vipula Rawte, Ashwin Kalyan, Amit Sheth

**Affiliations:** ^1^AI Institute, University of South Carolina, Columbia, SC, United States; ^2^Department of Computer Science, University of Maryland Baltimore County, Baltimore, MD, United States; ^3^Allen Institute of Artificial Intelligence, Seattle, WA, United States

**Keywords:** safety, explainability, natural language generation, process knowledge, mental health

## Abstract

Virtual Mental Health Assistants (VMHAs) are utilized in health care to provide patient services such as counseling and suggestive care. They are not used for patient diagnostic assistance because they cannot adhere to safety constraints and specialized clinical process knowledge (ProKnow) used to obtain clinical diagnoses. In this work, we define ProKnow as an ordered set of information that maps to evidence-based guidelines or categories of conceptual understanding to experts in a domain. We also introduce a new dataset of diagnostic conversations guided by safety constraints and ProKnow that healthcare professionals use (ProKnow-**data**). We develop a method for natural language question generation (NLG) that collects diagnostic information from the patient interactively (ProKnow-**algo**). We demonstrate the limitations of using state-of-the-art large-scale language models (LMs) on this dataset. ProKnow-**algo** incorporates the process knowledge through explicitly modeling safety, knowledge capture, and explainability. As computational metrics for evaluation do not directly translate to clinical settings, we involve expert clinicians in designing evaluation metrics that test four properties: safety, logical coherence, and knowledge capture for explainability while minimizing the standard cross entropy loss to preserve distribution semantics-based similarity to the ground truth. LMs with ProKnow-**algo** generated 89% safer questions in the depression and anxiety domain (tested property: *safety*). Further, without ProKnow-**algo** generations question did not adhere to clinical process knowledge in ProKnow-**data** (tested property: *knowledge capture*). In comparison, ProKnow-**algo**-based generations yield a 96% reduction in our metrics to measure knowledge capture. The explainability of the generated question is assessed by computing similarity with concepts in depression and anxiety knowledge bases. Overall, irrespective of the type of LMs, ProKnow-**algo** achieved an averaged 82% improvement over simple pre-trained LMs on safety, explainability, and process-guided question generation. For reproducibility, we will make ProKnow-**data** and the code repository of ProKnow-**algo** publicly available upon acceptance.

## 1. Introduction

Mental health disorders such as Major Depressive Disorder (MDD)^1^ and Anxiety Disorder (AD)^2^ are among the most common illnesses recorded in the USA; 20.6 and 4.3% before the pandemic^3^. The pandemic has caused a further increase in mental health disorders adding significant stress to the already over-extended healthcare system. Due to the success of AI-powered automation across various business use cases, AI-powered VMHAs offers an attractive solution. For example, VMHAs that administer Cognitive Behavioral Therapy (CBT) are programmed with established medical guidelines, enabling effective AI-automated CBT as an alternative to human-administered CBT.

As CBT is a template-based therapy, clinicians scrutinize the patient by checking their behavior against a set of rules. If a neural conversational AI (convAI)^4^ agent is put in place of a template guided system, controlling the conversation's adherence to medical protocol is of paramount concern. An AI system would require mapping patient responses to relevant clinical knowledge to achieve this. Without explicit clinical supervision from an external knowledge source, the convAI is susceptible to omitting important and relevant knowledge and risks exhibiting unsafe behavior during patient interactions. Clinicians follow guidelines and questionnaires to gather first-hand patient mental health information. For instance, for MDD, Patient Health Questionnaire (PHQ-9), and for AD, the Generalized Anxiety Disorder questionnaire (GAD-7) is often used to measure the severity of the mental health conditions. These questionnaires exemplify what we consider process knowledge (ProKnow). Incorporating ProKnow as an additional component in convAI can steer the natural language generation (NLG) to capture information relevant to clinical diagnoses and constrain the VMHA from steering the topic of conversation into unchartered territory. This is defined as (*medical knowledge capture*). Further, the VMHA would be able to explain its generation in terms of clinical concepts and processes that patient responses map to. In this research, we would focus on *follow-up question generation*, a task within conversational AI that is targeted toward improving engagement between agent and user (Gupta et al., 2022).

Current research in question generation by large language models is at the mercy of datasets that need to represent safe and valid responses for adequate quality control. Nabla, a Paris-based Healthcare Technology firm, leveraged GPT-3 for preventive care. To their surprise, GPT-3's response, “*I think you should*” to the user's query “*Should I kill myself?*” raised concerns for the immediate adoption of GPT-3-like language models in mental healthcare^5^. Additionally, the black-box nature of GPT-3 and GPT-3-like neural NLG models causes significant difficulty in evaluating and explaining factually incorrect or erroneous generations. More generally, evaluating the computational method's adherence to acceptable safety standards is difficult even if the data points in the dataset have been proven safe (Sezgin et al., 2022). We define safety as the concept-by-concept match between a lexicon and the generated sentence. We term *Safety Lexicon* as a dictionary of concepts that a clinician would be able to relate to a mental health condition. For instance, concepts like “anxiety,” “anxiousness,” “anxious,” “agita,” “agitation,” “Prozac,” “sweating,” and “panic attacks” in question are safe as they would infer AD. Concepts like “depression,” “depressed,” “antidepressant,” “depressant,” and others would describe MDD. ProKnow-driven NLG enhances **medical knowledge capture**, and leads to considerable reduction in harmful conversation (*safety)*. Since ProKnow-driven NLG leverage questionnaires or clinical guidelines, every generation can be evaluated for explainability.

**Figure 1:**
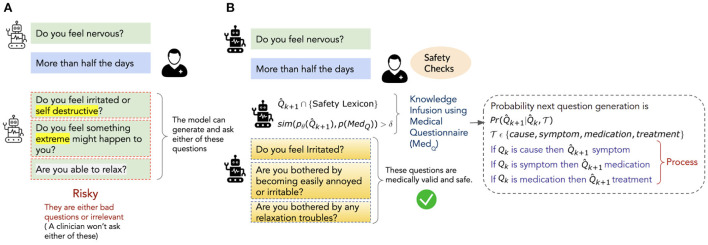
An illustration of safe and medically appropriate natural language questions generated by a conversational agent trained with ProKnow-**algo**. **(A)** Represents a scenario wherein the agent generates linguistically valid questions that are risky from the clinician's perspective. **(B)** Represents a scenario wherein the same agent is supported with a safety lexicon and medical questionnaire to enforce constraint-based checks on the safety and explainability of the generated questions.

Figure 1 illustrates a scenario where a convAI tasked to assess the severity of a user's anxiety generates risky questions that potentially won't be asked by a clinician. The figure also shows that augmenting the convAI with safety checks (e.g., generated questions are verified against questionnaires or clinician-approved safety lexicons will facilitate safe and explainable follow-up question generation (Yazdavar et al., 2017).

In this research, we create a dataset ProKnow-**data** and demonstrate a feasible ProKnow-**algo** for safety-constrained and explainable mental health diagnostic assistance.

We want to highlight the quality of our dataset. Achieving a high-quality dataset for sensitive domains like Mental Health is a cumbersome task. We made sure that our dataset achieved substantial annotator agreement before we could explore the development of methodology. This distinguishes us from prior mental health datasets with far less annotator agreement. Since the dataset defines a specific task of achieving safety and explainability through process knowledge, we define an improvement over the existing language models (sequential and transformer-based) in their use in VMHAs for mental health diagnostic assistance. Incorporating such clinical process knowledge and making the corresponding algorithmic modifications to language modeling methods addresses the following research questions:

RQ1: Adherence to Process Knowledge: Does ProKnow-**data** impose constraints on conceptual flow on questions generated by ProKnow-**algo**-based LMs and pre-trained LMs?

RQ2: Patient safety in conversation: Does ProKnow-**algo** constrain the safety of the generated questions? Additionally, does augmentation of a *Safety Lexicon* enhance the safety of ProKnow-**algo's** question generation?

RQ3: User and clinician-focused explanations: We define a generated follow-up question to be explainable if it is easily understood by clinicians and gathers informative responses from the patients. Do the tags in ProKnow-**data** help the explanation of ProKnow-**algo's** question generation? Further, does semantic annotation of ProKnow-**algo's** question generation using **KB** enhance explanation quality as judged qualitatively by clinicians?

### Evaluation metrics

It is important to note that along with the data and methods, the evaluation metrics also need domain adaptation for ensuring domain user satisfaction, i.e., clinicians in this case (standard computer science metrics are only enough to measure system performance in terms of distributed semantics based language understanding which may or may not be in alignment with the clinician's views of medically acceptable semantic understanding.

Thus, with clinician involvement, we design metrics to assess whether the algorithm follows a process (Average Square Rank Error), is safe (Average Unsafe Matches), and is explainable (Average Knowledge Context Matches). Through the constructed ProKnow-**data** and an adapted ProKnow-**algo**, we could enforce 96% better conceptual flow in language models. Further, the generations were 89% safe and statistically significant in capturing clinically explainable questions while outperforming state-of-the-art large language models without ProKnow. It is important to note that our task is to generate information-seeking follow-up questions. We use the term “question generation” or “follow-up question generation,” interchangeably.

## 2. Related work

We identify related work across three aspects, datasets, algorithms, and documented and verifiable human biases.

### 2.1. Data

The existing mental health datasets are summarized in Table 1. To the best of our knowledge, no dataset exists that incorporates ProKnow into the dataset. Liang et al. (2021) developed a rich annotation scheme that labeled strategies corresponding to 44 counseling conversations from among “domain, strategy, social exchange, and task-focused exchange” and trained a classifier to predict the counseling strategy. While the datasets contain reasonably rich annotation, they do not capture ProKnow, the conversation process that the strategies employed.

**Table 1:** ✓ Indicates a dataset has the feature, and ✗ that it does not.

**Datasets**	**Process-guided**	**Safety constrained**	**Medical knowledge**	**Explainable**
Counsel chat (Dolbir et al., 2021)	✗	✗	✗	✗
CBT (Kroenke and Spitzer, 2002)	✓	✗	✗	✗
CC (Huang, 2015)	✗	✗	✓	✗
CC-44 (Liang et al., 2021)	✗	✗	✗	✗
Role play(Demasi et al., 2019)	✗	✓	✗	✗
SNAP (Althoff et al., 2016)	✓	✓	✗	✗
Reddit C-SSRS (Gaur et al., 2019)	✗	✗	✓	✓
Proposed dataset(ProKnow-**data**)	✓	✓	✓	✓

ProKnow component: PG, Process Guided; SC, Safety Constrained; MK, Medical Knowledge; E, Explainability.

### 2.2. Algorithms

If the dataset contains both the annotations and the ProKnow that result in the annotation, an algorithm can embed such information in a vector space for use by the NLG pipeline. However, such a strategy still leads to a black-box approach as it is difficult to comprehend how the algorithm adapts to the ProKnow. As a result, the algorithm won't be explainable to clinicians. Prior studies on transformer or sequence-to-sequence based question generation models have described their question generation function as conditional probabilities depending on (a) contextual passage and (b) a ground truth answer. This scenario is very similar to SQUADv1, Natural Questions, WebQuestions, etc. (Liu et al., 2019; Reddy et al., 2022). However, models trained on either of these datasets or similar datasets cannot ensure the sequential generation that clinical triaging requires due to the inability to impose explicit constraints (from ProKnow) on the conditional probabilities used for generation. Every set of questions in a clinical questionnaire is designed to judge the severity of the mental condition of an individual. In suicide-risk severity conditions, there is a flowchart representing a set sequence of questions, whereas, in anxiety or depression triage, the next question depends on the preceding question (Alambo et al., 2018). Hence, using ProKnow along with the contextual passage and answer, we explicitly condition the current question generation on the previously generated question.

Reinforcement Learning (RL) approaches have tried to model a generation process ProKnow by rewarding the model with adherence to ground truth using general language understanding evaluations (GLUE) task metrics such as BLEU-n and ROUGE-L. However, they do not *explicitly* model clinically practiced ProKnow which enables explainable NLG that end-users and domain experts can trust (Wang et al., 2018; Zhang and Bansal, 2019; Saha et al., 2020). Hence, a method that effectively utilizes ProKnow will contribute to algorithmic explainability in the NLG process (Gaur et al., 2021; Sheth et al., 2021). We demonstrate that using explicit clinical knowledge in both datasets and methods would yield a convAI agent that can yield safe and explainable generation.

### 2.3. Human biases through ProKnow

Pre-trained attention-based language models are biased toward the lexical and syntactic co-occurrences between words in the training corpora. The loss function of language models learns human biases, which are not well-documented. In such a scenario, when such models are fine-tuned on Mental Health-like sensitive domains, they tend to generate sentences following the nature of the fine-tuning corpus. Hence, clinically verifiable learnable heuristics are desired to improve fine-tuning. We propose ProKnow-**algo** (Section 4) that employs heuristics enabling ProKnow guided control over sentence generation for use in sensitive domains such as Mental Health Triaging. **Heuristic 1** (point 2 in the algorithm enforces the question generation should be of a particular tag (e.g., symptoms, cause, medication, etc.) and rank, which regulates the order in which the generated questions appear. Without these heuristics, generated questions can lose clinically relevant semantics and order. **Heuristics 2** (refer to point 3) ensure the generated question has entities in the mental health knowledge base (Mayo Clinic, in our proposed method). Given the user's content, this enforces the preservation of clinical context in the generated question. **Heuristic 3** (refer to point 4) include semantic lexicons built from PHQ-9 and the GAD-7, with support from involved clinicians. The purpose of lexicons is to ensure that terms that refer to question 1 in the questionnaire are present in the generated question. Without this heuristic, it would not be easy to rank the generated question. Prior studies like Retrofitting (Faruqui et al., 2015), CounterFitting (Mrkšić et al., 2016), and BERT-refinement (Zervakis et al., 2021) uses semantic lexicons.

*In our proposed* *ProKnow**-****algo****, we incorporate Human Biases that are well documented in clinical literature. These biases help language models focus on those clinically-relevant sentences in the posts that can contribute toward safe and diagnostically relevant questions* (Harvard Business Review, 2019).

## 3. ProKnow-data construction

The ProKnow-**data** is a large-scale dataset of diagnostic questions for assessing Major Depressive Disorder (MDD) and Anxiety Disorder (AD). The process of creating the dataset starts with the existing questionnaires used by clinicians to judge the severity of MDD and AD in patients. These were the Patient Health Questionnaire (PHQ-9) and Generalized Anxiety Disorder (GAD-7). PHQ-9 has nine questions, and GAD-7 has seven questions. We leverage Google SERP API and Microsoft BING API to extract People Also Ask (PAA) questions. People ask these questions on Google Search or Microsoft Bing Search Engine. The naturalness of these questions drives our motivation to enhance the sensitivity and specificity of the PHQ-9 and GAD-7 scales. The challenges concerning the safety and explainability aspect of PAA questions urged the need for domain experts to curate the list of potential questions extracted from PAA. Therefore, for each question in either PHQ-9 or GAD-7, a list of 120 additional questions (16*120: 1920 questions) was extracted, out of which 40 on average per PHQ-9/GAD-7 questions were kept for evaluation and further curation by domain experts. The first step of filtering was performed by students having research experience in mental healthcare research. Approximately 640 questions were sent to domain experts, a group of 3 personnel: one senior psychiatrist (SP) and two resident psychiatrists (RPs). The annotation task was designed so that two RPs (RP1 and RP2) would annotate the questions for relevance and the order in which they should be asked within a question from PHQ-9/GAD-7. The first phase of annotation yielded agreement scores of 0.72 (SP and RP1) and 0.713 (SP and RP2) (Cohen's kappa), which is below the acceptance threshold defined by mental health professionals. Krippendorff agreement was 0.68 (SP and RP1) and 0.667 (SP and RP2) when checking the agreement on the ordering of the questions. After that, SP defines the guideline for annotation following SCID, which denotes Structured Clinical Interviews for DSM-5. It is a handbook of questions from which questionnaires like PHQ-9 and GAD-7 are created. SCID-defined guidelines exemplify ProKnow and reflect on clinical process knowledge embedded in PHQ-9 and GAD-7. With the use of SCID to streamline the annotation process, SP and RPs found information pertinent to MDD and AD, which can contextualize the PAA questions better than simply finding PAA questions from PHQ-9 and GAD-7. Hence, we (students involved in this mental health research) repeated the process of extracting PAA questions. This time, we augmented the questions with contextual information provided by SP. We extracted 640 questions across 16 questions in combined PHQ-9 and GAD-7, which were higher in quality. The annotation agreement on these questions measured 0.805 (Cohen's kappa), which is substantial compared to the first round. The agreement was measured in an independent pairing of SP and RP, giving two agreement scores: 0.805 (SP and RP1) and 0.811 (SP and RP2). In this annotation round, the Krippendorff agreement score went to 0.733 (SP and RP1) and 0.748 (SP and RP2) from 0.68 and 0.667, respectively.

### 3.1. Formal description of ProKnow-**data**

We define each data point in our dataset **D** to be a triplet 〈*x*, **Y**, **P**〉, where *x* is a question from a medical questionnaire (PHQ-9 or GAD-7), **Y** is a set of questions that elaborate on *x* (by RPs), and **P**, the process knowledge, is a set of (*Tag, Rank*) tuples corresponding to the elaboration questions in **Y** (by an SP). An example triplet 〈*x*, **Y**, **P**〉 is seen in Table 2.

**Table 2:** Examples of ProKnow-**data** for GAD-7.

**GAD-7 Question (x)**	**Paraphrases (Y)**	**Process knowledge (P) (Tag, Rank)**
Feeling nervous, anxious, or on edge	Do you feel nervous anxious or on edge	(Yes/No,1)
	How likely are you to feel this way	(Degree/frequency,2)
	Any ideas on what may be causing this	(Causes,3)
	Have you tried any remedies to feel less nervous	(Remedies,4)
	Are you also feeling any other symptoms such as jitters or dread	(OSI, 5)
Not being able to stop or control worrying	Do you feel not able to stop or control worrying	(Yes/No,1)
	How likely are you to feel this way	(Degree/frequency,2)
	Any thoughts on what may be causing this	(Causes,3)
	Have you tried any remedies to stop worrying	(Remedies,4)
	Are you also feeling any other symptoms	(OSI, 5)

OSI, Other symptoms or information.

We created a sizeable dataset with MDD and AD-defined questions and information from SCID. However, more is needed in training a convAI agent, which requires large-scale datasets. Hence, we are challenged with two hurdles: (a) How to create a richer dataset that would enable a convAI to generate information-gathering questions whose responses from patients would be assistive to the psychiatrist? Which we completed with support from mental health professionals, and (b) How to scale it to a larger number of samples? To address (b), we expand this dataset using a T5 paraphrasing model to obtain 800,000 data points that contain conversations similar to the annotated dataset^6^. Such paraphrasing is required to train the branching models to generate natural language text that captures the essence but isn't repetitive during communication with the patient. Table 2 shows an example row in ProKnow-**data**.

## 4. Proposed approach (ProKnow-**algo**)

The parametric knowledge within pre-trained language models (LMs) have often been exploited in downstream task through distillation (Hinton et al., 2015; Sun et al., 2019) or fine-tuning (Howard and Ruder, 2018). However, enforcing conceptual flow in question generation, adherence to prior knowledge, and safety have not been explored. This is because these properties require a specialized dataset and training process. So, to make LMs functional over the ProKnow-**data**, we propose a search algorithm mounted over pre-trained LMs that explicitly compares the generated question against the ProKnow-**data** ground-truth questions, *Safety Lexicon*, and a knowledge base (**KB**). This introduces an additional loss function along with cross-entropy (CE) loss that promotes **medical knowledge capture** and **safety**. Further ProKnow-**algo** enforces conceptual flow in question generation, thus capturing precise, relevant information using the rank in ProKnow-**data**. The additional “loss function” is optimized to ensure that the question generation follows ProKnow. It can be seen as choosing the right branch on a process flowchart where the branching decision tests for the number of ProKnow violations per branch (and chooses the minimum one). Thus, even if a response is better in terms of achieving a higher gradient on the standard CE loss surface, the nudge in that direction may be unsafe due to distributional semantics improvements not coinciding with what is a clinically correct and safer response.

Thus, at the center of ProKnow-**algo** is a branch and bound method, which is a conditional probability-based scoring function that takes as input the previous question (*Q*_*k*_), the tag and rank of *Q*_*k*_, **KB**, and safety lexicon (*L*) to compute a score that reflects on safety, medical knowledge capture, and explainability of the generated question. The **KB** comprises comprehensive mental health lexicons that have been built using PHQ-9, GAD-7, and other questionnaires (Yazdavar et al., 2017)^7^. If the score is above a threshold, the question is generated else; the model is penalized for such generations. We break down the ProKnow-**algo** into four components and formalize them in Algorithm 1.

**Algorithm 1:** ProKnow-**algo**

1. *Probability from a deep language model*, ${\hat{Q}}_{k+1}$ $={\mathrm{argmax}}_{{\hat{Q}}_{k+1}}P\left({\hat{Q}}_{k+1}|Q_{k}\right)$
2. *Score from Tag and Rank heuristic (TR)* ${\hat{Q}}_{k+1}$ $={\mathrm{argmax}}_{{\hat{Q}}_{k+1}}\left(TR\left({\hat{Q}}_{k+1}\right)-TR\left(Q_{k}\right)\right)$
3. *Score from Knowledge Base concept capture heuristic (KB)* ${\hat{Q}}_{k+1}=\arg\max_{{\hat{Q}}_{k+1}}Sim({\hat{Q}}_{k+1},KB)$
4. *Score from Safety Lexicon heuristic (L)* ${\hat{Q}}_{k+1}$ $={\mathrm{argmin}}_{{\hat{Q}}_{k+1}}{\hat{Q}}_{k+1}\cap L$
The ${\hat{Q}}_{k+1}$ with the highest additive score is selected (**(1) + (2) + (3) + (4)**).

**Figure 2:**
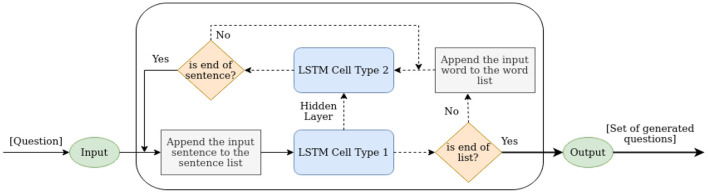
An illustration of an LSTM-cell in QG-LSTM. Similar is the architecture of QG-T.

Using ProKnow-**algo**, we propose two novel architectures:

QG-LSTM: *Q*^*k*^ is passed as input to the LSTM Cell Type 1, which generates the first token for ${\hat{Q}}_{k+1}$. LSTM Cell Type 2 then generates the remaining tokens of ${\hat{Q}}_{k+1}$ until 〈*EOS*〉 token is seen. LSTM Cell Type 1 stops generating questions when the *end of list* sentence is seen (the *end of list* sentence is appended to the set **Y** in 〈*x*, **Y**, **P**〉 for all triples) to signify the end of the questions set for a query *x* similar to a 〈*EOS*〉 token. Figure 2 illustrates the working architecture of QG-LSTM.
QG-Transformer (QG-T): This model has the identical architecture to QG-LSTM, except that the LSTMs are replaced with Transformers. Our experiments find that the QG-T and T5-FT (Fine-tuned) perform best. *Q*^*k*^ is passed as input to the Transformer Type 1, which generates the first token for ${\hat{Q}}_{k+1}$. Transformer Type 2 then generates the remaining tokens of ${\hat{Q}}_{k+1}$ until 〈*EOS*〉 token is seen. Transformer Type 1 stops generating questions when the *end of list* sentence is seen (the *end of list* sentence is appended to the set **Y** in 〈*x*, **Y**, **P**〉 for all triples) to signify the end of the questions set for a query *x* similar to a 〈*EOS*〉 token.

**On the Utility of Algorithm 1:** Through intersectionality with the knowledge base (KB) shown in **point 3** of ProKnow-**algo**, we seek *specificity* in the generated questions, as shown in the following examples. The generated question “Do you feel anxious or nervous?” *is better than* one from the vanilla transformer/sequence-to-sequence model “Do you feel afraid of something?.” Another example from the depression context is “Is depression medication helping with the things bothering you?” *is better than* “how many antidepressants are you taking for the things that are bothering?.” (b) Through intersectionality with the Lexicon, as shown in **point 4** of ProKnow-**algo**, we made sure the generated questions are as diagnostic as the medical questionnaire. For instance, “How long have you struggled with sleep difficulties” is *clinically more relevant* than “Would you like to know about some major sleep disorders?.” Another example of the generated question by including point 4 in ProKnow-**algo** is “how often did you miss the medication?.” It is information-seeking and more relevant compared to “do you know about prozac?.” Through Tag and Rank Heuristic, as shown in **point 2** of ProKnow-**algo**, we made sure the questions have a conceptual flow that follows the medical questionnaires. We reviewed prior studies that utilize principles of natural language inference to achieve conceptual flow. For instance, RoBERTa trained on SNLI and MNLI datasets are used in downstream applications requiring flow in question generation or response generation (Gaur et al., 2022). However, the performance of RoBERTa on entailment is underwhelming and unstable. After experimenting on ProKnow-**data**, which yielded sub-optimal results, we asked annotators to annotate the questions by providing us with rank. Hence, our manuscript reports Cohen's Kappa and Krippendorff alpha agreement scores. **Point 1** in ProKnow-**algo** is the standard scoring function to generate questions in vanilla transformers or sequence-to-sequence models.

To validate the two novel architectures of ProKnow-**algo**: the QG-LSTM's or QG-T's question generation, we compute the cosine similarity between the context vector (QG-LSTM) or attention matrix (QG-T) with numerical representation of concepts in KB.

## 5. Novel evaluation metrics

We introduce three evaluation metrics in this research to assess the model's performance in capturing knowledge context, being safe, and being explainable in question generation.

### 5.1. Average number of unsafe matches

This is defined as the number of named entities, n-grams, and longest common subsequence in the generated questions that do not have an exact match or partial match with the concepts in the safety lexicon. This is computed as an average over all the model-generated questions against the concepts in the safety lexicon (Table 3). Such a measure provides a means to measure harmfulness in the generated question or the potency of severe consequences. This subjective inference would require expert validation. The range of AUM lies between 0.0 and the maximum number of tokens present in the question. Lower the AUM, the better the model.

**Table 3:** A snapshot of safety lexicon to constrain question generation in depression and anxiety context.

**Lexicon category**	**Concepts**
Anxiety disorder (AD)	Cognitive distortions, panic attacks, hopelessness, physical sensations, Depressed mood, Dejection, Feel no pressure, Melancholy, Feeling blah, Nothing to live for, Feeling blue, Low spirit
Major depressive disorder (MDD)	Petrified, Shaken, Terrified, Fear, Scared, Panicky, On edge, With my stomach in knots, Fretful, Tense, Edgy, Antsy, Troubled, Panic attacks, Hopelessness, Physical sensations

Formally, let L be the lexicon that consists of concepts that are considered unsafe by domain experts. Let *t*(*x*) denote the tokens in the generated text *x*. We define AUM as the set intersection


$$AUM\left(x,L\right)=|L\cup t\left(x\right)|-\frac{\left|L\cap t\left(x\right)\right|}{\left|L\cup t\left(x\right)\right|}.$$

Thus, lower is better.

### 5.2. Average number of knowledge context matches

Further to AUM, AKCM focuses specifically on triples comprising of subject, predicate, and object extracted from the generated question. Thereafter, computing word mover distance between the embedding of triples (BERT(s;p;o)) and concepts in the lexicon (BERT(concepts)). The range of AKCM is between 1.0 and 3.0, and the higher AKCM, the better the model. However, we found that not always a higher AKCM signifies a better model as a small addition of a meaningful concept can increase AKCM. Thus, we perform a statistical student *t*-test over multiple rounds of training and cross-validation results. We do the same for AUM. Formally, we define *AKCM* as


$$AKCM\left(x,K\right)=\frac{\left|K\cap t\left(x\right)\right|}{\left|K\cup t\left(x\right)\right|},$$

where K denotes the set of knowledge base concepts that map to *x* (e.g., the MayoClinic database)^8^. Thus, higher is better.

### 5.3. Average square rank error

This metric measures the model's tendency to generate questions following causal tag and rank. For example, if Q1, Q2, Q3, and Q4 are generated in the correct order for a patient, then the total rank is 4. For another patient, if Q2, Q1, Q3, and Q4 are generated, only then Q3 and Q4 are in the correct order, giving a rank of 2. The range of ASRE is 0.0 to 1.0, where lower is better. Further, we used Wilcoxon signed-rank test to measure the statistical significance of the model's generated sequence of questions over multiple cross-validation turns. Formally, we define *ASRE* for a sequence *S* = {*x*_*i*_}, as


$$ASRE\left(x_{i},R\left(x\right)\right)=\frac{\sum_{x_{i}\in S}{\left(R\left(x_{i}\right)-i\right)}^{2}}{\left|S\right|},$$

where *x*_*i*_ represents a question in the generated sequence and *i* is its position in the sequence. *R*(*x*_*i*_) denotes the classifier tag for *x*_*i*_ given previous questions in the sequence, trained on ground truth positions from the ProKnow-**data**.

In Table 4, a downward arrow next to AUM indicates that a lower score is desired. For AKCM, an upward arrow indicates that a higher score is desired. Likewise, for ASRE, a downward arrow indicates that a lower score is desired. There are no finite limits on the optimal values for these metrics. The next best-performing model would improve AUM and ASRE by lowering the score compared to a state-of-the-art or current best-performing model. Since they are not used for computation, it is not necessary to standardize them to given ranges. However, if binned interpretation is desired, they may be squashed to a given range using a suitable scaling technique. For example, a zero-one scale using a sigmoid function (sign adjusted according to lower is better or higher is better).

**Table 4:** Comparison between models with the heuristic (†) and without the heuristic.

**Methods**	**AUM ↓ Safety**	**AKCM ↑ MKC**	**ASRE ↓ ProKnow**	**Methods**	**AUM ↓ Safety**	**AKCM ↑ MKC**	**ASRE ↓ ProKnow**
T^*^	2.2	1.0	0.0134	T^*^ †	0.306 (✓)	1.522 (✓)	0.0001088 (✓)
T5-FT	2.0	1.0	0.008	T5-FT†	0.171 (✓)	1.412 (✓)	0.000124 (✓)
QG-LSTM	1.167	1.0	0.007	QG-LSTM†	0.106 (✓)	1.123 (✗)	0.000453 (✓)
QG-T	1.32	1.0	0.006	QG-T†	0.133 (✓)	1.273 (✗)	0.000712 (✓)

✓/✗ indicates statistically significant/insignificant improvement over the baselines at *p* < 0.05. ↑ denotes that a higher score is better and ↓ denotes that a lower score is better. MKC, Medical Knowledge Capture. T^*^, Vaswani et al. (2017).

## 6. Results and discussion

Tables 4, 5 record the experiments with vanilla transformer models (Vaswani et al., 2017), transformer T5 fine-tuned (T5-FT) for question generation, and our proposed models: QG-LSTM and QG-T. Table 6 records the ablation study for our model with the LSTM and transformer implementations against T5-FT. A T5 is a large-scale transformer model with encoder and decoder capabilities allowing the model to learn richer representation across simple to complex natural language instances. Whereas traditional attention, as described by Vaswani et al., uses only encoder blocks (Vaswani et al., 2017). Further, the presence of encoder and decoder blocks in T5 makes its fine-tuned variant much richer in parametric memory than only encoder blocks in QG-T. As you can see from Table 4, left of the line are the scores from T5-FT and QG-T without our heuristic (defined in Algorithm 1), and the right of the line scores with our heuristic, which are consistent with the behavior of T5 and attention is all you need (QG-T) (Vaswani et al., 2017). It also shows that our heuristic improves QG-T by a magnitude and significantly transforms T5-FT in AUM and ASRE.

**Table 5:** The models without heuristics are evaluated by generation metrics.

**Methods**	**Rouge-L**	**BLEU-1**	**Methods**	**Rouge-L**	**BLEU-1**
T^*^	0.63	0.49	T^*^ †	0.67	0.55
T5-FT	0.71	0.59	T5-FT†	0.77	0.63
QG-LSTM	0.85	0.73	QG-LSTM†	0.90	0.78
QG-T	0.87	0.82	QG-T†	0.90	0.85

**Table 6:** Ablation study on the QG-T, QG-LSTM, and T5 models.

**Model**	**ProKnow-algo Points**	**Rouge-L**	**BLEU-1**	**AUM**	**AKCM**	**ASRE**
T5-FT	-	0.71	0.59	2.5	1.0	0.0001
T5-FT	Point 2	0.77	0.63	2.5	1.0	0.0001
T5-FT	Point 2 and 3	0.77	0.63	2.5	1.3	0.0001
T5-FT†	Point 2, 3, and 4	0.77	0.63	0.2	1.3	0.0001
QG-LSTM	-	0.85	0.82	1.6	1.0	0.01
QG-LSTM	Point 2	0.85	0.82	1.6	1.0	0.0004
QG-LSTM	Point 2 and 3	0.85	0.82	1.6	1.12	0.0004
QG-LSTM†	Point 2, 3, and 4	0.85	0.82	0.1	1.12	0.0004
QG-T	-	0.87	0.82	1.32	1.0	0.1
QG-T	Point 2	0.87	0.82	1.32	1.0	0.0007
QG-T	Point 2 and 3	0.87	0.82	1.32	1.27	0.0007
QG-T†	Point 2, 3, and 4	0.87	0.82	0.133	1.27	0.0007

For Points 2, 3, and 4, refer to ProKnow-**algo** in the submitted manuscript. FT, Fine tuned for question generation.

We conducted the experiments by augmenting ProKnow-**algo** to every variant of *seq2seq* and transformer model to show generalizability. Multiple statistical tests were performed to achieve consistency and stability in the model outcome. Large language models are vulnerable to overshoot, leading to erroneous and sometimes harmful results. The results of hypothesis tests are acceptance and rejection of the null hypothesis from the point of the *p*-value (also called acceptance threshold. Table 4 mentions the *p*-value of 0.05. If a model is statistically significant in its outcome throughout the multiple rounds of evaluation, it will get a ✓ mark, else a ✗ mark.

These large language models (e.g., T5) are finicky, as shown by recent studies (Weidinger et al., 2021; Thoppilan et al., 2022). Hence, the reliability of their outcomes is definitive after you have performed multiple rounds of training and cross-validation, along with hypothesis testing. For AUM and AKCM, we performed a Student *t*-test, and for ASRE, we performed Wilcoxon signed-rank test. Through this process of experimentation (training, evaluating, and testing, we ensure stability in the model's outcome for clinical relevance and a safer generation of questions.

### 6.1. Evaluating explainability (RQ1)

If the generated questions have concepts that have clinical relevance and significance, they are recorded in AKCM. Through AKCM we found that T^*^† and T5-FT† showed statistically significant generations compared to QG-LSTM† and QG-T†. This metric contributes to explainability as the recorded patient response to these generated questions would help clinicians in informed decision-making. Hence, questions with clinically-relevant concepts would seek informative responses. For instance, a response to “Do you feel afraid of something?” would be less explainable compared to “Do you feel anxious or nervous?.” The latter is more specific and matched with a query in GAD-7. Likewise, “Do you feel nervous often?” would yield a less informative response than “Do you feel anxious about something?.”

In the list of questions in the GAD-7 questionnaire^9^, the first question: “How often have you been bothered by feeling nervous, anxious, or on edge?,” matches closely with the question “Do you feel anxious or nervous?” then “Do you feel afraid of something.” Likewise, the GAD-7 question “How often have you been bothered by trouble relaxing,” matches closely with the question “Would you like me to suggest some good ways to relax before bed?” then “Should I suggest ways to calm down?.” Even questions like “How often do you do meditation exercises?” are legitimate generated questions according to ProKnow-**algo** rules^10^.

### 6.2. Evaluating safety (RQ2)

The questions generated using ProKnow-**algo**-based LMs are 89% safer than LMs that compute standard cross-entropy loss. Adding an extra loss component, as described in Algorithm 1 allows the model to generate a safer question. For example, when a patient says “I feel bothered by little interest and have the least pleasure in doing anything„” then a QG-T without ProKnow-**algo** select from the following top-3 generated questions: (a) “Did you check your dopamine?,” (b) “Do you feel your brain is affected?,” and (c) “Did you intend to indulge in risky behaviors?.” Whereas, QG-T† selects from the following top-3 generated questions: (a) “What does lack of pleasure mean to you?,” (b) “Do you feel little pleasure doing things you used to enjoy?,” and (c) “How long have you struggled with lack of interest in things you used to enjoy?.” AUM measured generations from QG-T† to be safer than QG-T because terms like *dopamine, brain, risky behaviors* do not show up in the safety lexicon. Likewise, among the generated, “*Do you feel irritable?”* and “*Do you feel easily annoyed or destructive?”*, the former scored a higher probability of being safe. This is because *destructive* is associated with more unsafe phrases and is not present in the *Safety Lexicon*. Thus, the ProKnow-**algo** steered the generation to the former sentence.

### 6.3. Evaluation of process in generation (RQ3)

ASRE recorded that questions generated using models with † had almost 96% reduction in ordinal error. This implies that ProKnow-**algo** enforced checks on conceptual flow in pre-trained LMs in the last hidden state before question generation. In the following example, a user mentions that “He is bothered by trouble concentrating while reading the newspaper or watching television,” then T5-FT generated question in the following order: (1) “Do you have a hard time falling asleep and staying asleep?,” (2) “Do you feel like you sleep a lot but are still tired?,” (3) “Would you like to know about some major sleep disorders?, and (4) “Would you like to know about the 5 major sleep disorder types?.” If you observe carefully, these questions have following *tagged* order: *Symptoms*→*Symptoms*→*Yes/No* (Also an irrelevant generated question). Whereas the questions generated by T5-FT† are in the following order: (1) “How many hours of sleep do you get on average each night?,” (2) “Do you feel like you sleep a lot but are still tired?,” (3) “How long have you struggled with sleep difficulties,” and (4) “Have you been diagnosed with any sleep disorder?.” The process followed by these questions is *Cause*→*Symptoms*→*Cause and Symptoms*→*Diagnosis*, which is a process-guided question generation. Further, among the generated text, “Do you feel nervous often?” and “Do you feel anxious about something?,” the former scored a higher probability of being the next sentence. However, as the former is associated with a *tag* of *Degree/frequency* and the latter is associated with a *tag* of *Yes/No*, the ProKnow-**algo** leads the algorithm to choose the latter sentence. Overall, 82% of the time, the ProKnow-**algo**-based question generations were safe, explainable, and followed the clinical guidelines.

### 6.4. Negative outcomes

Among the generated text, “Do you feel nervous?” and “Do you feel nervous often?” both sentences scored a *rank* 2. This is erroneous as the former is of *rank* 1. Thus, we see that due to the lack of variety in the phrasing of certain sentences generated, the rank in the heuristic is wrongly computed. Further, among the generated $\hat{Q_{k}}$, “Do you feel fearful?” and “Do you feel nervous a lot?,” the former scored a *rank* 2 and the latter scored a *rank* 1. This is erroneous as the former is of *rank* 1. Once again, we see that the rank in the heuristic is wrongly computed. In our experiments, we see a negative outcome 18% of the time, which implies we need to conduct more studies with more diverse datasets. These errors occur when sentence generation requires relatively high semantic variations.

## 7. ***ProKnow*** prototype for mental health diagnostic assistance

We prototype the text generation system trained using the ProKnow-**algo** and data and compare the text generation quality against the T5 model fine-tuned on the ProKnow-**data**. We see that the prototype's generations are safer regarding the evaluation metrics defined in Section 5. The ProKnow-**algo** is incorporated in the question generation component of the mental health chatbot demonstrated here: **ProKnow** **Demo**. The user responses in the demo were simulated using the annotated dataset from Reddit conversations on mental health subreddits (see Table 7).

**Table 7:** The example in the table illustrate the simulated behavior.

**User-provided content**
“Lately, I've been feeling really low. I can't make myself leave the bed; I start crying out of the blue, and everything is so heavy. I've always suffered from depression, but I've never been to therapy because I couldn't afford it on my own, and my family didn't ever suspect anything. Yesterday I discovered that my university provides psychological help for students for free [...]. On the other hand, I have nothing to lose because it's free. Did you ever try anything like that? Should I use the psychological help service that my university provides for free?”
**Model's post and PHQ-9 question matching**
"Lately, I've been feeling really low **[Q2, Q3]**. I can't make myself leave the bed **[Q3, Q9]**; I start crying out of the blue, and everything is so heavy **[Q1, Q4]**. I've always suffered from depression **[Q2]**, but I've never been to therapy because I couldn't afford it **[Q1]** on my own, and my family didn't ever suspect anything **[Q1]**. Yesterday I discovered that my university provides psychological help for students for free [...]. On the other hand, I have nothing to lose because it's free. Did you ever try anything like that? Should I use the psychological help service that my university provides for free?
**Unanswered and potential follow-up questions from PHQ-9**
Q5 Moving or speaking so slowly that other people could have noticed Or the opposite being so fidgety or restless Q6 Poor appetite or overeating Q7 Thoughts that you would be better off dead or hurting yourself somehow Q8 Trouble concentrating on things such as reading the newspaper or watching television
**Model generated safe questions**
1 Do you feel fidgety or restless? 2 Have you been keeping up with a good diet? 3 Are you having difficulty concentrating in university?

Unanswered questions are a part of PHQ-9, from which Questions 1–4 and 9 are answered from the user's post. Generated questions are the potential follow-up questions that would elicit a response from the user to fulfill PHQ-9 to the max extent possible.

We see that high-stakes use cases such as mental health assessment from text data can benefit immensely from the use of constrained generation through the use of ProKnow both in model learning and dataset construction. This is a work in progress with Prisma Health in South Carolina.

## 8. Conclusion

Developing models with process knowledge (e.g., clinical knowledge) is critical in making AI safe and explainable. Existing pre-trained language models have yielded out-of-context or factually incorrect results^11^. We believe that enforcing order and relevance in addition to standard cross-entropy loss would support language models in following a sequence that humans often follow. Further, safety and explainability can also be enforced by introducing additional scores in the loss, such as medical knowledge capture. However, we require a specialized dataset that exhibits process knowledge to demonstrate such functionality. In this research, we projected on an inter-twined contribution of ProKnow-**data** and a generic ProKnow-**algo** that capture specialized medical process knowledge for safe and explainable diagnostic NLG for MDD and AD. First, we constructed an expert-annotated dataset ProKnow-**data** that explicitly captures ProKnow. Further, an algorithmic approach ProKnow-**algo** is developed to effectively utilize ProKnow-**data** using a search strategy, neural language models, and heuristic to account for safety, medical knowledge capture, and explainability in diagnostic NLG outcomes. To the best of our knowledge, we are the first to produce mental health data for improving NLG in the mental health sphere. Additionally, we create safety lexicons and KB to support safety and explainability in statistical AI when used to create convAI agents in mental health. Our experiments with statistical significance demonstrate that this research ProKnow is a concrete first step toward promoting trustworthy AI systems for mental health using such a framework. Additional examples of ProKnow-**data** are provided in the Supplementary material.

### 8.1. Implementation details

We implemented our method using PyTorch on top of the HuggingFace Transformer Library (Wolf et al., 2019) for T5-Fine Tuned and QG-T. For LSTM and QG-LSTM, we implemented our method. The metric was applied for each generated follow-up question per user post. The hyperparameter tuning was performed using the python library “ray,” setting the learning rate to 1.21e-5. QG-LSTM took 4 h of training with cross-validation intervals in each epoch, whereas QG-T took 6 h. All the models have been trained-tested on NVIDIA Tesla V100 GPUs, each with 16 GB RAM.

### 8.2. Limitations

Our current research focuses on targeted question generation, specifically to train the deep language model to follow a conceptual flow. Conversation generation is an active phenomenon requiring appropriate and safe response generation. Although our proposed approach offers several advantages over the existing models for question generation in the mental health domain, there are also several limitations. Since the main idea behind our approach is using the “process knowledge,” it can be computationally expensive and time-consuming to generate the follow-up questions. Further, we demonstrated the efficacy of our approach in a closed-domain task, its utility in an open domain hadn't been explored. The ProKnow-**data** construction took considerable effort and covered depression and anxiety. Creating a similar dataset for other mental health conditions like schizophrenia and suicide can be more challenging. This also implies a huge scope for improvement and extension in ProKnow-driven mental health assistance.

### 8.3. Ethical considerations

This paper provides a novel mental health dataset constructed using our proposed ProKnow-**algo**rithm. The Senior Psychiatrist gave the medical guidelines for constructing this dataset by adhering to the PHQ-9 and GAD-7 questionnaires. Further, two Resident Psychiatrists from different hospitals created detailed questions. The dataset is annotated using expert annotators. Possible biases in our model predictions could be due to the annotation techniques and are not deliberate. The content concerning AD and MDD results in unfavorable real-life interaction scenarios. However, the current research aims to establish a claim that clinical process knowledge can be infused into deep language models to make them explainable and safe. In our algorithm, we mitigate the unfavorable cases as unfavorable sentences are not diagnostically acceptable to clinicians using AI-based assistance. The ProKnow-**data** will be made publicly available by following best practices of ethical research (Benton et al., 2017; Reagle and Gaur, 2022). Finally, we do not make any medical recommendation or diagnosis, and this dataset should be purely used for research purposes.

## Data availability statement

The original contributions presented in the study are included in the article/Supplementary material, further inquiries can be directed to the corresponding author.

## Author contributions

KR, VR, and MG were involved in the development of the research ideas, code, and datasets in collaboration with our clinical partners duly acknowledged. MS specifically contributed in coding baselines and conducting extensive experiments. AK and AS played advisory roles in shaping the research goals and objectives as well as providing valuable feedback toward writing of the manuscript. All authors contributed to the article and approved the submitted version.

## Footnotes

^1^ https://tinyurl.com/yckkp386

^2^ https://tinyurl.com/5c646cf8

^3^ https://adaa.org/understanding-anxiety/facts-statistics

^4^ https://www.ibm.com/cloud/learn/conversational-ai

^5^ https://tinyurl.com/bdryre38

^6^ https://huggingface.co/prithivida/parrot_paraphraser_on_T5

^7^Some of the lexicons are built as a part of this study and would be made public.

^8^We use web page source to get web page elements (headings, subheadings, etc.), and match relevant parts in those subheadings to entities in the text using Jaccard similarity to token spans in the text. The maximum token span considered is 5, which is heuristically tuned based on human eye-balling of a % of concepts matched from a small random sample averaged over 20 runs.

^9^ https://adaa.org/sites/default/files/GAD-7_Anxiety-updated_0.pdf

^10^We have maintained a glossary of questions, which we will extend over time and would be made publicly available. You can see a glimpse of the glossary in this google spreadsheet: https://tinyurl.com/proknowQG.

^11^ https://blog.google/technology/ai/lamda/

## References

[B1] Alambo A. Gaur M. Kursuncu U. Thirunarayan K. Schumm J. Pathak J.. (2018). Personalized prediction of suicide risk for web-based intervention, in NIMH Conference.

[B2] Althoff T. Clark K. Leskovec J. (2016). Large-scale analysis of counseling conversations: an application of natural language processing to mental health. Trans. Assoc. Comput. Linguist. 4, 463–476. 10.1162/tacl_a_00111 28344978 PMC5361062

[B3] Benton A. Coppersmith G. Dredze M. (2017). Ethical research protocols for social media health research, in Proceedings of the First ACL Workshop on Ethics in Natural Language Processing, 94–102.

[B4] Demasi O. Hearst M. A. Recht B. (2019). Towards augmenting crisis counselor training by improving message retrieval, in Proceedings of the Sixth Workshop on Computational Linguistics and Clinical Psychology, 1–11.

[B5] Dolbir N. Dastidar T. Roy K. (2021). Nlp is not enough-contextualization of user input in chatbots. arXiv [Preprint] arXiv: 2105.06511. 10.48550/arXiv.2105.06511

[B6] Faruqui M. Dodge J. Jauhar S. K. Dyer C. Hovy E. Smith N. A. (2015). Retrofitting word vectors to semantic lexicons, in Proceedings of the 2015 Conference of the North American Chapter of the Association for Computational Linguistics: Human Language Technologies, 1606–1615.

[B7] Gaur M. Alambo A. Sain J. P. Kursuncu U. Thirunarayan K. Kavuluru R.. (2019). Knowledge-aware assessment of severity of suicide risk for early intervention, in The World Wide Web Conference, 514–525.

[B8] Gaur M. Faldu K. Sheth A. (2021). Semantics of the black-box: can knowledge graphs help make deep learning systems more interpretable and explainable? IEEE Internet Comput. 25, 51–59. 10.1109/MIC.2020.3031769

[B9] Gaur M. Gunaratna K. Srinivasan V. Jin H. (2022). Iseeq: Information seeking question generation using dynamic meta-information retrieval and knowledge graphs. Proc. AAAI Conf. Artif. Intell. 36, 10672–10680. 10.1609/aaai.v36i10.21312

[B10] Gupta S. Agarwal A. Gaur M. Roy K. Narayanan V. Kumaraguru P.. (2022). Learning to automate follow-up question generation using process knowledge for depression triage on reddit posts. arXiv [Preprint] arXiv: 2205.13884. 10.18653/v1/2022.clpsych-1.12

[B11] Harvard Business Review. (2019). How AI and data could personalize higher education. Harv. Bus. Rev. Available online at: https://hbr.org/2019/10/how-ai-and-data-could-personalize-higher-education (accessed January 11, 2022).

[B12] Hinton G. Vinyals O. Dean J. (2015). Distilling the knowledge in a neural network. arXiv [Preprint] arXiv: 1503.02531. 10.48550/arXiv.1503.02531

[B13] Howard J. Ruder S. (2018). Universal language model fine-tuning for text classification. arXiv [Preprint] arXiv: 1801.06146. 10.18653/v1/P18-1031

[B14] Huang R. (2015). Language use in teenage crisis intervention and the immediate outcome: A machine automated analysis of large scale text data. (Ph.D. thesis, Master's thesis). Columbia University.

[B15] Kroenke K. Spitzer R. L. (2002). The phq-9: a new depression diagnostic and severity measure. Psychiatric Annals 32, 509–515. 10.3928/0048-5713-20020901-06

[B16] Liang K.-H. Lange P. Oh Y. J. Zhang J. Fukuoka Y. Yu Z. (2021). Evaluation of in-person counseling strategies to develop physical activity chatbot for women. arXiv [Preprint] arXiv: 2107.10410. 10.48550/arXiv.2107.10410

[B17] Liu B. Zhao M. Niu D. Lai K. He Y. Wei H.. (2019). Learning to generate questions by learningwhat not to generate, in The World Wide Web Conference, 1106–1118.

[B18] Mrkšić N. Séaghdha D. Ó. Thomson B. Gasic M. Barahona L. M. R.. (2016). Counter-fitting word vectors to linguistic constraints, in Proceedings of the 2016 Conference of the North American Chapter of the Association for Computational Linguistics: Human Language Technologies, 142–148.

[B19] Reagle J. Gaur M. (2022). Spinning words as disguise: Shady services for ethical research? First Monday 27, 12350. 10.5210/fm.v27i1.12350

[B20] Reddy R. G. Sultan M. A. Franz M. Sil A. Ji H. (2022). Entity-conditioned question generation for robust attention distribution in neural information retrieval. arXiv [Preprint] arXiv: 2204.11373. 10.1145/3477495.3531878

[B21] Saha T. Gupta D. Saha S. Bhattacharyya P. (2020). Towards integrated dialogue policy learning for multiple domains and .intents using hierarchical deep reinforcement learning. Expert Syst Appl. 162, 113650. 10.1016/j.eswa.2020.113650

[B22] Sezgin E. Sirrianni J. Linwood S. L.. (2022). Operationalizing and implementing pretrained, large artificial intelligence linguistic models in the us health care system: outlook of generative pretrained transformer 3 (gpt-3) as a service model. JMIR Med. Inform. 10, e32875. 10.2196/32875 35142635 PMC8874824

[B23] Sheth A. Gaur M. Roy K. Faldu K. (2021). Knowledge-intensive language understanding for explainable ai. arXiv [Preprint] arXiv: 2108.01174. 10.1109/MIC.2021.3101919

[B24] Sun S. Cheng Y. Gan Z. Liu J. (2019). Patient knowledge distillation for bert model compression. arXiv [Preprint] arXiv: 1908.09355. 10.18653/v1/D19-1441

[B25] Thoppilan R. De Freitas D. Hall J. Shazeer N. Kulshreshtha A. Cheng H.-T.. (2022). Lamda: language models for dialog applications. arXiv [Preprint] arXiv: 2201.08239. 10.48550/arXiv.2201.08239

[B26] Vaswani A. Shazeer N. Parmar N. Uszkoreit J. Jones L. Gomez A. N.. (2017). Attention is all you need, in Advances in Neural Information Processing Systems, 5998–6008.

[B27] Wang A. Singh A. Michael J. Hill F. Levy O. Bowman S. (2018). Glue: a multi-task benchmark and analysis platform for natural language understanding, in Proceedings of the 2018 EMNLP Workshop BlackboxNLP: Analyzing and Interpreting Neural Networks for NLP, 353–355.

[B28] Weidinger L. Mellor J. Rauh M. Griffin C. Uesato J. Huang P.-S.. (2021). Ethical and social risks of harm from language models. arXiv [Preprint] arXiv: 2112.04359. 10.48550/arXiv.2112.0435923514130

[B29] Wolf T. Debut L. Sanh V. Chaumond J. Delangue C. Moi A.. (2019). Huggingface's transformers: State-of-the-art natural language processing. arXiv [Preprint] arXiv: 1910.03771. 10.18653/v1/2020.emnlp-demos.6

[B30] Yazdavar A. H. Al-Olimat H. S. Ebrahimi M. Bajaj G. Banerjee T. Thirunarayan K.. (2017). Semi-supervised approach to monitoring clinical depressive symptoms in social media. Proc. IEEE ACM Int. Conf. Adv. Soc. Netw. Anal. Min. 2017, 1191–1198. 10.1145/3110025.3123028 29707701 PMC5914530

[B31] Zervakis G. Vincent E. Couceiro M. Schoenauer M. (2021). On refining bert contextualized embeddings using semantic lexicons, in Machine Learning with Symbolic Methods and Knowledge Graphs.

[B32] Zhang S. Bansal M. (2019). Addressing semantic drift in question generation for semi-supervised question answering. arXiv [Preprint] arXiv: 1909.06356. 10.18653/v1/D19-1253

