# Supplementary material for: ProKnow: Process knowledge for safety constrained and explainable question generation for mental health diagnostic assistance
Source: Front Big Data. 2023 Jan 9;5:1056728. doi: 10.3389/fdata.2022.1056728 (PMC9869802; doi:10.3389/fdata.2022.1056728)
Supplement: Supplementary file 1 [file Presentation_1.pdf]

The guide for using the ‘Multicell Transformer Architecture’ implementation

By Misagh Soltani

Description:

The file next to this document is an implementation of the ‘Multicell Transformer Architecture’ in Python programming language. This document is to explain how to initialize and use the modules using custom settings and have a good understanding if you want to make any changes.

To read about basic Transformers[1] architecture visit this link.

Table of Contents:

DESCRIPTION **1**

GENERAL OVERVIEW **2**

INPUTS & OUTPUTS **3**

REQUIREMENTS **3**

- PYTHON 3 3
- PYTORCH LIBRARY 3
- TORCHTEXT LIBRARY 4
- CUDA TOOLKIT 4
- GENSIM LIBRARY 4
- IGNITE LIBRARY 4

MODULES DESCRIPTIONS **4**

- MODULES TO IMPORT 4
- FUNCTIONS 5
  - *get_data_info(data_dict)* *5*
  - *get_key_by_value(input_dict, input_val)* *5*
  - *get_token(sentence, vocab2ix)* *6*
  - *preprocess_data(data_path, save_path)* *6*
  - *preprocess_test_data(data_path, save_path)* *7*
  - *decode_sentences(sentence_sets, vocab2ix)* *8*
  - *create_embedding_layer(vocab2ix, w2v_model, save_path, device)* *8*
  - *print_predictions(sent_lst)* *9*
  - *beam_search_decoder(post, k, device)* *9*
- CLASSES 9
  - *class Transformer(*args, **kwargs)* *9*
  - *class SentencesDataset(*args, **kwargs)* *12*
  - *class multilevelTransformer (*args, **kwargs)* *13*
  - *class PositionalEncoding (*args, **kwargs)* *15*
  - *class TransformerEncoderLayer (*args, **kwargs)* *15*
  - *class TransformerDecoderLayer (*args, **kwargs)* *16*
  - *class TransformerEncoder (*args, **kwargs)* *18*
  - *class TransformerDecoder (*args, **kwargs)* *18*

---

[1] Polosukhin, Illia; Kaiser, Lukasz; Gomez, Aidan N.; Jones, Llion; Uszkoreit, Jakob; Parmar, Niki; Shazeer, Noam; Vaswani, Ashish (2017-06-12). "Attention Is All You Need". arXiv: 1706.03762

HOW TO RUN **19**

PREPARING THE REQUIREMENTS & DATA 19
INITIALIZING & USING THE MULTILEVEL TRANSFORMER MODEL 20
PREDICTING QUESTIONS 21
EVALUATING THE MODEL USING TEST DATA 21

General Overview

The goal of this architecture is to predict a set of questions based on a sentence. In this architecture, we introduce a new cell that consists of two Transformers. The first cell (we call it Transformer Type 1) gets a sentence as input and returns a word as output, and the second cell (we call it Transformer Type 2) gets a word as input, which is the output of the first cell, and also returns a word as output. The bigger cell (the cell containing two Transformers) gets a sentence as input and returns a set of sentences as output.

Note: The two transformers are different cells that both have all the regular recurrent and standard steps of their own.

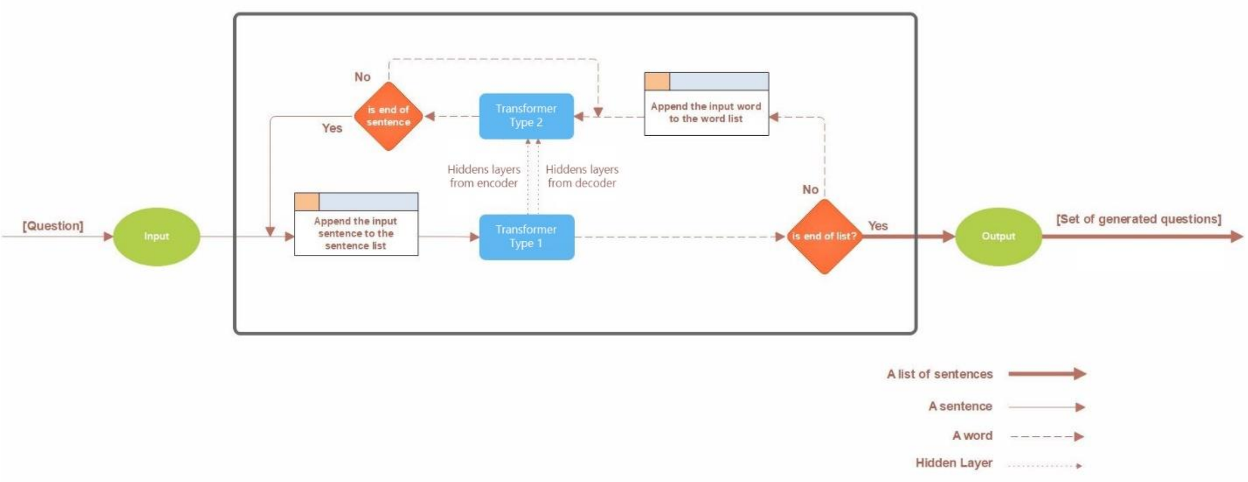

Figure 1 - The general overview diagram showing the interconnections of two Transformer cells used in multicell architecture

Now we explain the functionality of the bigger cell and the interconnections between two inner cells.

Figure 1 shows an overview diagram of how and by what structure two Transformers are connected to work together in order to generate the final output of the outer cell. As it is shown, a sentence is passed to the outer cell as input. When the input comes into the outer cell it will be passed through the Transformer type 1 which predicts a word to its output. Then the output of cell type 1 (which is a word) is passed through cell type 2 which will return a word. While the output word of cell type 2 is not the end of the sentence tag (in our implementation <EOS>), the output of this cell will be used as input to itself. If we get the end of the sentence tag from the output of cell type 2, we will make a sentence consisting of the predicted words so far, and use it as the input of cell type 1. Another data transmitting between two cells is the encoder and decoder's hidden layer weights of cell type 1 which is used as the initial encoder and decoder's hidden layers of cell type 2.

This procedure will continue to execute until we have a set of sentences which are the final output of the outer cell.

Inputs & Outputs

So far, we have a general understanding of the architecture. Now we will explain what the word and sentence shape inputs and outputs are.

Note: in this specific implementation we have a dataset of some patients whom each has a set of sentences and some questions that can be predicted from those sentences.

As shown in ***Figure 1***, for the multilevel Transformer, we have sentence-set outputs, each predicted from its corresponding input sentence.

The following is an example of the inputs and outputs of the model.

Input (a sentence):

0. *Feeling nervous, anxious, or on edge*

Output (a set of questions):

1. *Do you feel nervous anxious or on edge?*
2. *Yes, I do.*
3. *How likely are you to feel this way?*
4. *I sometimes feel this way.*
5. *Any ideas on what may be causing this?*
6. *No ideas.*
7. *Have you tried any remedies to feel less nervous?*
8. *Not at all.*
9. *Are you also feeling any other symptoms such as jitters or dread?*
10. *No, never.*

The input sentence (i.e. sentence number 0) is passed to the multilevel Transformer. Inside this architecture, the input sentence is routed to Transformer type 1, which will generate the first word of the first question of the output (i.e. question number 1) – i.e. the word '*Do*'. Then this word will be routed to cell type 2 to predict the following word of the same question (i.e. question number 1) - i.e. the word '*you*'. This process will continue until we reach the end of the question (i.e. when the word '*edge*' is generated). At this point, the whole question (i.e. question number 1) is created and passed to cell type 1 to generate the first word of the next sentence (i.e. sentence number 2). All of these steps will continue until the last sentence (i.e question number 10) is generated. Finally, the multilevel Transformer will return all the generated sentences (i.e. questions in number 1 to number 10 if the answers are given by a user) as output.

Requirements

Python 3 [1]:

This architecture implementation is coded in Python programming language, version 3.

- Check this link to install Python 3.

PyTorch Library [2]:

PyTorch is an optimized tensor library for deep learning using GPUs and CPUs.

[1] https://www.python.org/download/releases/3.0/
[2] https://pytorch.org/

We have used PyTorch tensors and functions throughout the project.

- Check this link to install PyTorch

TorchText Library [1]:

This library is part of the PyTorch project.

We have used the bleu_score() function of this library to compute the BLEU Score in the evaluation step.

- Check this link to install TorchText

CUDA Toolkit [2]:

CUDA (an acronym for Compute Unified Device Architecture) is a parallel computing platform and application programming interface (API) model created by Nvidia. It allows software developers and software engineers to use a CUDA-enabled graphics processing unit (GPU) for general-purpose processing, an approach termed GPGPU (general-purpose computing on graphics processing units). The CUDA platform is a software layer that gives direct access to the GPU's virtual instruction set and parallel computational elements, for the execution of compute kernels.[3]

If you want to get the advantage of executing computations on GPU, you have to install this toolkit.

- Check this link to install CUDA Toolkit

Gensim Library [4]:

Gensim is a free open-source Python library for representing documents as semantic vectors, as efficiently (computer-wise) and painlessly (human-wise) as possible.

We use this library to run embedding models to use in the architecture embedding layers.

- Check this link to install Gensim

Ignite Library [5]:

Ignite is a high-level library to help with training and evaluating neural networks in PyTorch flexibly and transparently.

We will use the functions prepared for computing RougeL in this library in the evaluation step.

- Check this link to install ignite.

Modules Descriptions

In this section, we will explain all the important classes and functions implemented to construct the desired architecture.

Modules to import

```
from gensim.models import Word2Vec, KeyedVectors
from torch.utils.data import Dataset, DataLoader
from torchtext.data.metrics import bleu_score
```

---

[1] https://pytorch.org/text/stable/index.html#module-torchtext
[2] https://developer.nvidia.com/cuda-toolkit
[3] https://en.wikipedia.org/wiki/CUDA
[4] https://radimrehurek.com/gensim/
[5] https://pytorch.org/ignite/

```python
from ignite.metrics import RougeL
import torch.nn as nn
import torch
import pickle
import re
import time
import datetime
import os
```

Functions

get_data_info(data_dict)

This function gets the data and returns some information about it. This information contains the maximum length of sentences and questions in the data, the maximum number of sentences corresponding to a patient in the dataset, the maximum number of questions that can be predicted from the sentences mentioned before, the number of unique words in the dataset, and a dictionary mapping each unique word to an integer.

Input:

data_dict: a nested dictionary that has integers as keys, whereas each number represents a patient. The value mapped to each key is a dictionary whose keys are sentences for that particular patient, and the value for each of these keys is a list of questions that can be predicted from that specific sentence.

Outputs:

vocab2ix: a dictionary mapping each unique word in the dataset to an integer

n: the number of unique words in the dataset in integer

max_q_num: the maximum number of sentences related to a patient in the dataset in integer

max_gen_num: the maximum number of questions (excluding the answers count) that can be predicted from the sentences in integer

max_word_num: the maximum of the length of all the sentences and questions in the dataset.

get_key_by_value(input_dict, input_val)

This function gets a dictionary and a value as input then returns the corresponding key of that value in that dictionary.

Inputs:

input_dict: a dictionary containing input_val with a specific key.

input_val: the value of the key for finding in the input_dict

Output:

The key in input_dict which the input_val is mapped to

get_token(sentence, vocab2ix)

This function gets a sentence and a dictionary mapping each unique word in the dataset to an integer then returns a list of numbers that each is the integer value present in vocab2ix mapped to the corresponding word of the sentence.

Before splitting the sentence and mapping each word to the related integers, the sentence is edited using regular expressions so it will only contain the characters A-Z, a-z, 0-9, " ' ", and space ("\s"), then the end of sentence tag ("<EOS>") is added to its tail.

Inputs:

sentence: a sentence in string

vocab2ix: a dictionary mapping each unique word in the dataset to an integer

Output:

A tensor in float type, with the shape of (Number of substrings in sentence string separated using space after the sentence is edited and the <EOS> tag is added to end of it. Each index of this tensor contains the numbers that each is the integer value present in vocab2ix mapped to the corresponding word of the edited sentence.

preprocess_data(data_path, save_path)

This function gets the path to the dataset (which is in the form of nested dictionaries) to load the data from. After loading the data, it prepares the input data for training each of the two types of transformers used in the architecture. The data for each cell is stored in two variables, one for the x values (i.e. inputs) and the other for the y values (i.e. outputs).

The x data for cell type 1 is a list of all possible sequences using the sentences and their corresponding predicted questions and answers of all patients. The y data for this cell is the first word of the first question related to that sentence if the last sentence of the sequence of the x data in the same index has come from the sentences (not questions). If not, it is the first word of the next question or answer as if there is another question after that one. Finally, if there are not any questions following that, it will be a padding tag (<PAD>). Also, the sequence length is the maximum number of questions and answers that can be predicted from the sentences.

In the same way, the x data for cell type 2 is a list of all possible sequences using the words of the predicted questions and answers of all patients. The y data for this cell is the following word of the last word of the sequence of the x data in the same index if it is not the last word of its related question. If so, it is the <EOS> tag. Also, if the last word of the sequence of the x data in the same index is the <EOS> tag, its corresponding y data will be <PAD> tag. And the sequence length is the maximum of the length of all the sentences and questions in the dataset.

These data will be saved in the local storage for faster loading.

Inputs:

data_path: the path to a directory on local storage including the dataset in *.pkl format and the mentioned nested dictionary structure.

save_path: the path to a directory on local storage where the preprocessed data for the two cells should be saved.

Outputs:

The output of this function is a dictionary containing the keys below and their corresponding values.

x_data1: a tensor in float type, containing all possible sequences using the sentences and their corresponding predicted questions of all patients. Sequences are set of PyTorch tensors that each item is the token number of the word in the same position of the string forms. The shape of this tensor is (number of patients * max_q_num * sequence length of cell type 1, sequence length of cell type 1 (2 * max generatable questions num +1: because of the question answers), Maximum number of words + 1 for <EOS> tag)

- Note that the first sequence for each sentence will be a sequence with the sentence for the first item, and all <PAD> tags for other items. The second sequence for each sentence will be a sequence with the sentence for the first item, the first question for the second item, and all <PAD> tags for other items. This will continue until for each sentence we have the last sequence with the sentence for the first item, the first question for the second item, the second question for the third item, …, the nth question for the $(n + 1)^{th}$ items, etc.)

y_data1: a tensor in float type, containing corresponding predicted the first word of the next sentence for all possible sequences using the sentences and questions for all patients. This tensor is a set of numbers, each number is the token number of the word. The shape of this tensor is (number of patients * max_q_num * sequence length of cell type 1)

x_data2: a tensor in float type, containing all possible sequences using the words of the predicted questions of all patients. Sequences are set of PyTorch tensors that each item is the token number of the word in the same position of the string forms. The shape of this tensor is (number of patients * max_q_num * sequence length of cell type 1, sequence length of cell type 2 (this is because we will train cell type 2 right after cell type 1 for each of its sequences), sequence length of cell type 2)

y_data2: a tensor in float type, containing corresponding predicted next word of each words sequence for all possible sequences using the words of questions for all patients. This tensor is a set of numbers, each number is the token number of the word. The shape of this tensor is (number of patients * max_q_num * sequence length of cell type 1, sequence length of cell type 2 (this is because we will train cell type 2 right after cell type 1 for each of its sequences))

data_info_dict: a dictionary of the data information returned from the get_data_info() function

preprocess_test_data(data_path, save_path)

This function gets the path to the test dataset (which is in the form of nested dictionaries) to load the data from. After loading the data, it prepares the input data for evaluating the multicell transformer architecture. The data is stored in two variables, one for the x values (i.e. inputs) and the other for the y values (i.e. outputs).

The x data is a list of all sentences of all patients. The y data of index i is the questions and answers that should be predicted from the sentence of index i.

The data will be saved in the local storage for faster loading in the future.

Inputs:

data_path: the path to a directory on local storage including the dataset in *.pkl format and the mentioned nested dictionary structure.

save_path: the path to a directory on local storage where the preprocessed data for the evaluation should be saved.

Outputs:

The output of this function is a dictionary containing the keys below and their corresponding values.

x_test_lst: a list of sentences in string type. The shape of this list is (number of patients * max_q_num)

y_test_lst: a list of sets of questions in string type. The shape of this list is (number of patients * max_q_num, 2 * max_gen_num + 1)

data_info_dict: a dictionary of the data information returned from the get_data_info() function

decode_sentences(sentence_sets, vocab2ix)

This function gets some sets of sentences that are tokenized using token numbers in vocab2ix, then returns the sentences converted from number tokens to words (Also removes the tags).

Inputs:

sentence_sets: this is a list with the shape of (number of input sentences to the model, number of questions generated + 1 (sentence), maximum sentence length) where the first index is the input sentence of the model and other indices are questions.

vocab2ix: a dictionary mapping each unique word in the dataset to an integer.

Outputs:

str_sentence_sets: a list of strings, where the value of the first index is the string of the input sentence and the rest is strings of generated questions by the model.

lst_sentence_sets: a list of sets of sentences and questions in word tokenized form.

create_embedding_layer(vocab2ix, w2v_model, save_path, device)

This function gets the embedding model and vocabulary dictionary and creates an embedding layer to use in Transformers. This layer is also saved on local storage for faster loading.

Inputs:

vocab2ix: a dictionary mapping each unique word in the dataset to an integer.

w2v_model: the embedding model to convert each word into vectors. All embedding models can be used, but they should be in keyed vector format.

save_path: the path to where the embedding layer will be saved after being created.

device: a *torch.device* variable which specifies which device (CPU or GPU) is the created layer in.

Output:

Embedding_layer: a *torch.nn.Embedding*, which is the embedding layer to be used as the first layer of the Transformer sequences.

print_predictions(sent_lst)

This function gets a set of string sentences and prints them to the screen. The first sentence and the rest which have been predicted from the first sentence, are distinguished.

Input:

sent_list: list of strings to be printed.

Output:

This function has no outputs and only prints the list on the screen.

beam_search_decoder(post, k, device)

This function gets a set of probabilities of each word in sequence and returns k number of the best sequences based on them.

Input:

post: the posterior of network. The shape of *post* is (batch_size, seq_length, vocab_size).

k: beam size of decoder.

device: a *torch.device* object representing the device on which a *torch.Tensor* is or will be allocated. The *torch.device* contains a device type ('cpu' or 'cuda') and an optional device ordinal for the device type.

Output:

indices: a beam of index sequence. The shape of indices is (batch_size, beam_size, seq_length).

log_prob: a beam of log likelihood of sequence. The shape of log_probs is (batch_size, beam_size).

Classes

class Transformer(**args, **kwargs*)

This class is inherited from *torch.nn.Module* [1] - the base class for all neural network modules. Each of the two types of cells used in this architecture is an instance object of this class. Here you can add and initialize any other layers from nn.Module such as *nn.DropOut* [2], etc to use in each Transformer. We have already used some layers including *nn.LogSoftmax* [3] as we will use Negative Log Loss Likelihood in the training section.

This class is the Pytorch's default implementation with a few changes. For more details, see the PyTorch's documentation[4].

[1] https://pytorch.org/docs/stable/generated/torch.nn.Module.html
[2] https://pytorch.org/docs/stable/generated/torch.nn.Dropout.html
[3] https://pytorch.org/docs/stable/generated/torch.nn.LogSoftmax.html
[4] https://pytorch.org/docs/stable/generated/torch.nn.Transformer.html

Constructor

Here we initialize the parent class of *Transformer* class and all the attributes of the class.

Inputs:

ntoken: the number uniqe words in our vocabulary.

seq_len: the length of the sequence given to the model.

d_model_src: the number of expected features of each item in our sequence in input of the encoder.

d_model_tgt: the number of expected features of each item in our sequence in input of the decoder.

nhead: the number of heads in the multiheadattention models (this is set to 1 in decoder)

num_encoder_layers: the number of sub-encoder-layers in the encoder.

num_decoder_layers: the number of sub-decoder-layers in the decoder.

dim_feedforward: the dimension of the feedforward network model.

dropout: the dropout value.

activation: the activation function of encoder/decoder intermediate layer, relu or gelu.

custom_encoder: custom encoder.

custom_decoder: custom decoder.

layer_norm_eps: the eps value in layer normalization components.

embedding_layer: a *nn.Embedding* [1], which is a simple lookup table that stores embeddings of a fixed dictionary and size.

embedding_dim: the number of features used to represent each word.

batch_first: if `True`, then the input and output tensors are provided as (batch, seq, feature). If `False` (seq, batch, feature).

device: a *torch.device* [2] object representing the device on which a *torch.Tensor* [3] is or will be allocated. The *torch.device* contains a device type ('cpu' or 'cuda') and an optional device ordinal for the device type.

Methods

Transformer.forward(self, src, target, h_enc0 = None, h_dec0 = None, src_mask = None,
tgt_mask = None, memory_mask = None, src_key_padding_mask = None,
tgt_key_padding_mask = None, memory_key_padding_mask = None)

this method is used for the forward pass step during the training process and also to make predictions.

[1] https://pytorch.org/docs/stable/generated/torch.nn.Embedding.html#torch.nn.Embedding
[2] https://pytorch.org/docs/stable/tensor_attributes.html#device-doc
[3] https://pytorch.org/docs/stable/tensors.html

Inputs:

src: the sequence to the encoder. The shape of src is (batch size, sequence length for that type of transformer, number of words in each sequence item * embedding dimension) (required).

target: the sequence to the decoder. The shape of target is (batch size, target sequence length for that type of transformer, number of words in each sequence item * embedding dimension) (required).

h_enc0: the initial weights of the hidden layer of the encoder. The shape of h_enc0 is (batch_size, dim_feedforward). (required in transformer type 1)

h_dec0: the initial weights of the hidden layer of the decoder. The shape of h_dec0 is (batch_size, target sequence length, dim_feedforward). (required in transformer type 1)

src_mask: the additive mask for the *src* sequence (optional).

tgt_mask: the additive mask for the *tgt* sequence (optional).

memory_mask: the additive mask for the encoder output (optional).

src_key_padding_mask: the *ByteTensor* mask for src keys per batch (optional).

tgt_key_padding_mask: the *ByteTensor* mask for tgt keys per batch (optional).

memory_key_padding_mask: the *ByteTensor* mask for memory keys per batch (optional).

Outputs:

output: the output of the transformer for that step of the forward pass. The *output* variable is a list of probabilities of the output word to be any of the words in the vocabulary dictionary. The shape of *out* is (batch size, vocabulary size)

h_enc: the weights of the hidden layer of the encoder at the end of that step. The shape of *h_enc* is (batch_size, dim_feedforward).

h_dec: the weights of the hidden layer of the decoder at the end of that step. The shape of *h_dec* is (batch_size, target sequence length, dim_feedforward).

Transformer. generate_square_subsequent_mask(self, sz)

This method generate a square mask for the sequence. The masked positions are filled with float('-inf'). Unmasked positions are filled with float(0.0).ons.

Inputs:

sz: the size of the square mask (required).

Outputs:

mask: a square mask for the sequence.

class SentencesDataset(*args, **kwargs)

This class is inherited from *torch.utils.data.Dataset* [1] - An abstract class representing a dataset that will be used in training and evaluating steps. We initialize objects of this class using the preprocessed data saved in variables or local storage.

Constructor

Here we initialize the attributes whether they are in a local file or a variable and whether the dataset is to use in the training phase or evaluation phase.

Inputs:

device: a *torch.device* object representing the device on which a *torch.Tensor* is or will be allocated. The *torch.device* contains a device type ('cpu' or 'cuda') and an optional device ordinal for the device type.

is_test (default = False): if True, specifies that the instance object will be used in the evaluation, otherwise it will be used in the training phase.

Methods

SentencesDataset.from_dict(self, data_dict)

This method gets preprocessed data from a dictionary and sets the data as dataset values.

Inputs:

data_dict: the dictionary from which the dataset will load the data. For training data we have data_dict = { 'x_data1': x_data1, 'y_data1': y_data1, 'x_data2': x_data2, 'y_data2': y_data2, 'data_info': data_info_dict } and for the test data we have data_dict = { 'x_test': x_test_lst, 'y_test': y_test_lst, 'test_data_info': test_data_info_dict }

Outputs:

This method has no outputs.

SentencesDataset.from_file(self, data_dict)

This method loads preprocessed data from a file on local storage and sets the data as dataset values.

Inputs:

data_path: the path to *.pt file from which the dataset will load the data. For training data we have data files with the following structure. data_dict = { 'x_data1': x_data1, 'y_data1': y_data1, 'x_data2': x_data2, 'y_data2': y_data2, 'data_info': data_info_dict } and for the test data we have data_dict = { 'x_test': x_test_lst, 'y_test': y_test_lst, 'test_data_info': test_data_info_dict }

Outputs:

This method has no outputs.

SentencesDataset.get_data_info(self)

This method returns the information of data used as the dataset.

Inputs:

This method has no inputs.

[1] https://pytorch.org/docs/stable/data.html#torch.utils.data.Dataset

Outputs:

self.data_info: a dictionary containg information about data. E.g. data_info_dict = { 'vocab2ix': vocab2ix, 'vocab_size': vocab_size, 'max_q_num': max_q_num, 'max_gen_num': max_gen_num, 'max_word_num': max_word_num }

class multilevelTransformer (**args, **kwargs*)

This class is the main class of this architecture. We can say this is the implementation of the diagram shown in ***Figure 1*** and the main part that manages the interconnections of two cell types. You should declare an object from this class, then train it on your train data and use the model. Or load the pre-trained model and use it.

Constructor

Here we prepare our model to train or make use of.

Inputs:

dim_feedforward: the dimension of the feedforward network model.

embedding_layer: a *nn.Embedding*, which is a simple lookup table that stores embeddings of a fixed dictionary and size. This layer will be used as the first layer of each of two inner Transformers.

data_info_dict: a dictionary of the data information returned from the *get_data_info()* function.

embedding_dim: the number of features used to represent each word.

num_encoder_layers: the number of sub-encoder-layers in the encoder.

num_decoder_layers: the number of sub-decoder-layers in the decoder.

nhead: the number of heads in the multiheadattention models (this is set to 1 in decoder)

dropout: the dropout value.

device: a *torch.device* object representing the device on which a *torch.Tensor* is or will be allocated. The *torch.device* contains a device type ('cpu' or 'cuda') and an optional device ordinal for the device type.

Methods

multilevelTransformer.train(self, train_dataset, batch_size, num_epochs, criterion1, criterion2, optimizer1, optimizer2)

This method is used to train the multilevel Transformer model on training data using the desired settings.

Inputs:

train_dataset: an object of *SentencesDataset* class containing the prepared data for training the model.

batch_size: the number of training examples utilized in one iteration.

num_epochs: a hyperparameter that defines the number of times that the learning process will work through the entire training dataset.

criterion1: a PyTorch loss function [1] used in the training process for inner Transformer type 1. In this implementation, we use the negative log-likelihood loss (nn.NLLLoss [2]).

criterion2: a PyTorch loss function used in the training process for inner Transformer type 2. In this implementation, we use the negative log-likelihood loss (nn.NLLLoss).

optimizer1: an optimizer object from PyTorch optimization package - *torch.optim* [3] - used in the training process for inner Transformer type 1. In this implementation, we use the Adam optimizer [4].

optimizer2: an optimizer object from PyTorch optimization package - *torch.optim* - used in the training process for inner Transformer type 2. In this implementation, we use the Adam optimizer.

Outputs:

This method has no outputs.

multilevelTransformer.evaluate(self, test_dataset)

This method is used to evaluate the trained model on the test dataset using BLEU Scores and RougeL scores.

Inputs:

test_dataset: an object of *SentencesDataset* class containing the prepared data for evaluating the model.

Outputs:

scores_dict: a dictionary containing two dictionaries, one for BLEU scores and another for RougeL scores.

multilevelTransformer.save_model(self, path)

This method is used to save the trained model and its attributes to the local storage in *.pth format for future use.

Input:

path: a string path to the directory where the model should be saved.

Outputs:

This method has no outputs.

multilevelTransformer.load_model(self, path)

This method is used for the forward pass step during the training process and also to make predictions.

Input:

path: a string path to the *.pth file that the model should be loaded from.

Outputs:

This method has no outputs.

---

[1] https://pytorch.org/docs/stable/nn.html#loss-functions
[2] https://pytorch.org/docs/stable/generated/torch.nn.NLLLoss.html#torch.nn.NLLLoss
[3] https://pytorch.org/docs/stable/optim.html#module-torch.optim
[4] https://pytorch.org/docs/stable/generated/torch.optim.Adam.html#torch.optim.Adam

multilevelTransformer.predict(self, sentence_set)

this method is used to predict the questions from input sentences.

Input:

sentence_set: a list of string sentences from which the questions will be predicted.\

beam_size: the number of beams used in beam search when looking for the best sequence.

Output:

x: a PyTorch tensor containing predicted questions for each input sentence from sentence_set. Each item of this tensor is a set of predicted questions in the format of a tensor of number tokens. x has a shape of (number sentences in sentence_set, beam_size, maximum number of prediction questions, maximum number of words in predictions) – note: in this implementation we just return the first beam's result.

class PositionalEncoding (**args, **kwargs*)

This class is inherited from *torch.nn.Module* and is used to get the positional encodings of the word embeddings.

Constructor

Inputs:

d_model: the dimension of the input word embeddings.

dropout: the dropout value.

max_len: the maximum length of the sequence to be positionally encoded.

Methods

PositionalEncoding.forward(self, x)

This method gets the word sequential word embeddings and returns the positionally encoded embeddings.

Inputs:

x: the word embeddings to be encoded. The shape of *x* is (batch_size, seq_len, embedding_dim)

Outputs:

Positionally encoded word embeddings. The shape of output is (batch_size, seq_len, embedding_dim)

class TransformerEncoderLayer (**args, **kwargs*)

TransformerEncoderLayer is made up of self-attn and feedforward network. This standard encoder layer is based on the paper "Attention Is All You Need". Ashish Vaswani, Noam Shazeer, Niki Parmar, Jakob Uszkoreit, Llion Jones, Aidan N Gomez, Lukasz Kaiser, and Illia Polosukhin. 2017. Attention is all you need. In Advances in Neural Information Processing Systems, pages 6000-6010. Users may modify or implement in a different way during application.

This class is the Pytorch's default implementation with a few changes. For more details, see the PyTorch's documentation[1].

Constructor

Inputs:

d_model: the number of expected features in the input (required).

nhead: the number of heads in the multiheadattention models (required).

seq_len: the length of the sequence used in encoder.

dim_feedforward: the dimension of the feedforward network model.

dropout: the dropout value.

activation: the activation function of intermediate layer, relu or gelu.

layer_norm_eps: the eps value in layer normalization components.

batch_first: If `True`, then the input and output tensors are provided as (batch, seq, feature).

device: a *torch.device* object representing the device on which a *torch.Tensor* is or will be allocated. The *torch.device* contains a device type ('cpu' or 'cuda') and an optional device ordinal for the device type.

Methods

TransformerEncoderLayer.forward(self, x)

Inputs:

src: the sequence to the encoder layer (required).

h_enc0: the initial weights of the hidden layer of the encoder. The shape of h_enc0 is (batch_size, dim_feedforward). (required in transformer type 1)

src_mask: the mask for the src sequence (optional).

src_key_padding_mask: the mask for the src keys per batch (optional).

Outputs:

src: the result of the encoder layer. The shape of output is (batch_size, seq_len, embedding_dim)

h_enc_n: the weights of the hidden layer of the encoder at the end of that step. The shape of *h_enc_n* is (batch_size, dim_feedforward).

class TransformerDecoderLayer (**args, **kwargs*)

TransformerDecoderLayer is made up of self-attn, multi-head-attn and feedforward network. This standard decoder layer is based on the paper "Attention Is All You Need". Ashish Vaswani, Noam Shazeer, Niki Parmar, Jakob Uszkoreit, Llion Jones, Aidan N Gomez, Lukasz Kaiser, and Illia

[1] https://pytorch.org/docs/stable/generated/torch.nn.TransformerEncoderLayer.html

Polosukhin. 2017. Attention is all you need. In Advances in Neural Information Processing Systems, pages 6000-6010. Users may modify or implement in a different way during application.

This class is the Pytorch's default implementation with a few changes. For more details, see the PyTorch's documentation[1].

Constructor

Inputs:

d_model: the number of expected features in the input (required).

nhead: the number of heads in the multiheadattention models (required).

dim_feedforward: the dimension of the feedforward network model.

dropout: the dropout value.

activation: the activation function of intermediate layer, relu or gelu.

layer_norm_eps: the eps value in layer normalization components.

batch_first: If `True`, then the input and output tensors are provided as (batch, seq, feature).

device: a *torch.device* object representing the device on which a *torch.Tensor* is or will be allocated. The *torch.device* contains a device type ('cpu' or 'cuda') and an optional device ordinal for the device type.

Methods

TransformerDecoderLayer.forward(self, x)

Inputs:

tgt: the sequence to the decoder layer (required).

memory: the sequence from the last layer of the encoder (required).

h_dec0: the initial weights of the hidden layer of the decoder. The shape of *h_dec0* is (batch_size, target sequence length, dim_feedforward). (required in transformer type 1)

tgt_mask: the mask for the tgt sequence (optional).

memory_mask: the mask for the memory sequence (optional).

tgt_key_padding_mask: the mask for the tgt keys per batch (optional).

memory_key_padding_mask: the mask for the memory keys per batch (optional).

Outputs:

tgt: the result of the decoder layer. The shape of output is (batch_size, seq_len, embedding_dim)

h_dec_n: the weights of the hidden layer of the decoder at the end of that step. The shape of *h_dec_n* is (batch_size, target sequence length, dim_feedforward).

[1] https://pytorch.org/docs/stable/generated/torch.nn.TransformerDecoderLayer.html

class TransformerEncoder (*args, **kwargs)

TransformerEncoder is a stack of N encoder layers.

This class is the Pytorch's default implementation with a few changes. For more details, see the PyTorch's documentation[1].

Constructor

Inputs:

encoder_layer: an instance of the *TransformerEncoderLayer()* class (required).

num_layers: the number of sub-encoder-layers in the encoder (required).

norm: the layer normalization component (optional).

Methods

TransformerEncoder.forward(self, x)

Inputs:

src: the sequence to the encoder (required).

h_enc0: the initial weights of the hidden layer of the encoder. The shape of h_enc0 is (batch_size, dim_feedforward). (required in transformer type 1)

mask: the mask for the src sequence (optional).

src_key_padding_mask: the mask for the src keys per batch (optional).

Outputs:

output: the result of the encoder layer. The shape of *output* is (batch_size, seq_len, embedding_dim)

h_enc: the weights of the hidden layer of the encoder at the end of that step. The shape of *h_enc* is (batch_size, dim_feedforward).

class TransformerDecoder (*args, **kwargs)

TransformerDecoder is a stack of N decoder layers.

This class is the Pytorch's default implementation with a few changes. For more details, see the PyTorch's documentation[2].

Constructor

Inputs:

decoder_layer: an instance of the *TransformerDecoderLayer()* class (required).

num_layers: the number of sub-decoder-layers in the decoder (required).

norm: the layer normalization component (optional).

---

[1] https://pytorch.org/docs/stable/generated/torch.nn.TransformerEncoder.html
[2] https://pytorch.org/docs/stable/generated/torch.nn.TransformerDecoder.html

Methods

TransformerDecoder.forward(self, x)

Inputs:

tgt: the sequence to the decoder (required).

memory: the sequence from the last layer of the encoder (required).

h_dec0: the initial weights of the hidden layer of the decoder. The shape of h_dec0 is (batch_size, target sequence length, dim_feedforward). (required in transformer type 1)

tgt_mask: the mask for the tgt sequence (optional).

memory_mask: the mask for the memory sequence (optional).

tgt_key_padding_mask: the mask for the tgt keys per batch (optional).

memory_key_padding_mask: the mask for the memory keys per batch (optional).

Outputs:

output: the result of the decoder layer. The shape of *output* is (batch_size, seq_len, embedding_dim)

h_dec: the weights of the hidden layer of the decoder at the end of that step. The shape of *h_dec* is (batch_size, target sequence length, dim_feedforward).

How to Run

Preparing the Requirements & Data

There are some requirements to prepare for using the model.

One of the requirements is to load or create an embedding model in keyed vector format to use when creating the embedding layer. Below is an example that loads google's pre-trained word2vec model from local storage using the Gensim library.

```
model_w2v =
KeyedVectors.load_word2vec_format('.\model\w2v_model\GoogleNews-vectors-
negative300.bin', binary = True)
```

The next is to preprocess the raw data or load the preprocesses data from a file. The below is the code for loading the raw data file from the specified folder. The data will be preprocessed and then saved to the path passed to function and also returned as a variable.

```
# load the raw data from file & preprocess and save it
 data_dict = preprocess_data('./data/dataset.pkl',
'./data/preprocessed_data.pt')
```

Then we can initialize our training dataset object from the returned dictionary or a file which preprocessed and saved before.

```
# create a SentenceDataset object to load the training data
train_dataset = SentencesDataset(device, is_test = False)
# load the data to dataset from file
train_dataset.from_file('./data/preprocessed_data.pt')
# load the data to dataset from a dictionary
train_dataset.from_dict(data_dict)

data_info = train_dataset.get_data_info()
```

We also specify our device to use all over the code. Below is an example of how to use GPU if it is available and otherwise, use CPU.

```
# specify the device
device = torch.device('cuda' if torch.cuda.is_available() else 'cpu')
```

In this step, you should either create an embedding layer (which will automatically be saved to the file) or load existing from the file. Below is an example of both methods.

```
# create & save and get an embedding layer
embedding_layer = create_embedding_layer(data_info['vocab2ix'],
model_w2v, './model/embedding_layer.pt', device)
# load an embedding layer already existing in local storage
embedding_dict = torch.load('./model/embedding_layer.pt', map_location
= device)
embedding_layer = nn.Embedding(data_info['vocab_size'],
embedding_dict['embedding_dim'], padding_idx = 0).to(device)
embedding_layer.load_state_dict(embedding_dict['state_dict'])
embedding_layer.eval()
```

Initializing & Using the Multilevel Transformer Model

Now it is time to create an instance object of the *multilevelTransformer* class. In this step, you can set the hidden size and the number of recurrent layers of the model.

```
num_encoder_layers = 5
num_decoder_layers = 5
dropout = 0.1
hidden_size = 300
num_epochs = 5
batch_size = 10
nhead = 10
lr = 0.001
multiTransformer = MultiLevelTransformer(hidden_size, embedding_layer,
data_info, embedding_dict['embedding_dim'], num_encoder_layers,
num_decoder_layers, nhead, dropout, device)
```

Now you can either create and train a new model with desired loss functions, optimizers, and other settings, use it or save it for future use, or load the existing model from the file. See the below example.

```
# train and save the model using customized configuration
criterion1 = nn.NLLLoss()
optimizer1 = torch.optim.Adam(multiTransformer.model1.parameters(), lr)
criterion2 = nn.NLLLoss()
optimizer2 = torch.optim.Adam(multiTransformer.model2.parameters(), lr)
multiTransformer.train(train_dataset, batch_size, num_epochs,
criterion1, criterion2, optimizer1, optimizer2)
# save the trained model to file
multiTransformer.save_model('./model/model_state.pth')
# load an existing model from file
multiTransformer.load_model('./model/model_state.pth')
```

Predicting Questions

Now that everything is ready and you have an already trained model, you can pass a set of sentences to the model's *predict*() function, to predict the questions set. Below is an example of how to get the result, convert from number tokens to word tokens and print them properly.

```
sentence_set = ['Feeling afraid, as if something awful might happen',
'Feeling nervous, anxious, or on edge']
res = multiTransformer.predict(sentence_set)
# convert predictions from number tokens to word tokens
res_str, _ = decode_sentences(res, multiTransformer.vocab2ix)
print_predictions(res_str)
```

Evaluating the Model Using Test Data

Then preprocess the raw test data or load the preprocesses test data from a file. The below is the code for loading the raw test data file from the specified folder. The test data will be preprocessed and then saved to the path passed to function and also returned as a variable.

```
# load the raw data from file & preprocess and save it to file
test_data_dict = preprocess_test_data('./data/dataset.pkl',
'./data/preprocessed_test_data.pt')

# create a SentenceDataset object to load the test data
test_dataset = SentencesDataset(device, is_test = True)
# load the test data to dataset from file
train_dataset.from_file('./data/preprocessed_data.pt')
# load the test data to dataset from a dictionary
train_dataset.from_dict(data_dict)
data_info = train_dataset.get_data_info()
```

The last step is to evaluate the model.

```
# evaluate the model using the test dataset
```

```
multiTransformer.evaluate(test_dataset)
```

The evaluation scores will be logged into '@current_directory/log/evaluation_log.pt', so you can load and print them on the screen using the code below.

```
directory = './log/evaluation_log.pt'
print(f'Loading the scores from "{directory}".')
scores_dict = torch.load(directory)
BLEU_score = scores_dict['bleu_scores']
Rouge_L = scores_dict['rouge_l']
print(f'Loading the scores from "{directory}": Done')
print(f'\nDataset BLEU Scores:\n\tBLEU-1 = {BLEU_score["bleu-1"]:.4f}\n\tBLEU-2 = {BLEU_score["bleu-2"]:.4f}\n\tBLEU-3 = {BLEU_score["bleu-3"]:.4f}\n\tBLEU-4 = {BLEU_score["bleu-4"]:.4f}')
print(f'Dataset ROUGE-L Scores:\n\tROUGE-L-P = {Rouge_L["Rouge-L-P"]:.4f}\n\tROUGE-L-R = {Rouge_L["Rouge-L-R"]:.4f}\n\tROUGE-L-F = {Rouge_L["Rouge-L-F"]:.4f}')
```
